# Selective recovery effects of short-term beetroot juice supplementation after exercise-induced muscle damage in trained female volleyball players

**DOI:** 10.1080/15502783.2026.2751785

**Published:** 2026-10-20

**Authors:** Shuning Zhang, Xudong Yang, Hongqi Xu, Qing Liu, Helong Quan

**Affiliations:** a Exercise Capacity Assessment and Promotion Research Center, School of Physical Education, Northeast Normal University, Changchun, Jilin, People's Republic of China; b China Volleyball College, Beijing Sport University, Beijing, People's Republic of China; c Department of Sport Science, Chungnam National University, Daejeon, South Korea; d Jilin Green Food Engineering Research Institute, Changchun, Jilin, People's Republic of China

**Keywords:** Dietary nitrate, exercise-induced muscle damage, recovery, volleyball, female athletes

## Abstract

**Background:**

Beetroot juice (BRJ) has been proposed as a nutritional aid for recovery from exercise-induced muscle damage (EIMD), but evidence in female team-sport athletes is limited. It also remains unclear whether any benefit extends across functional, pain-related, swelling, and strength outcomes.

**Methods:**

Seventeen female university volleyball players entered a randomized, double-blind, placebo-controlled crossover trial. Four did not complete the second period, so outcomes were analyzed with linear mixed models. After baseline testing, participants performed 200 loaded vertical jumps and consumed BRJ or placebo at 24, 48, and 72 h post-EIMD. Recovery outcomes were countermovement jump (CMJ) height, volleyball-specific agility time, pressure pain threshold, thigh circumference, visual analog scale soreness, and knee isokinetic measures. Holm-adjusted planned contrasts were used.

**Results:**

CMJ height was the clearest BRJ-related functional finding. The treatment × time interaction showed a trend toward statistical significance (*P* = 0.062), and CMJ height was higher under BRJ than PLA at 72 h post-EIMD (*P* = 0.033, d = 1.01). Calf pressure pain threshold also favored BRJ at 72 h, accompanied by a significant treatment × time interaction (*P* < 0.001). In contrast, agility time and soreness changed over time without clear between-condition differences, while thigh pressure pain threshold, thigh circumference, and low-velocity knee isokinetic outcomes showed no consistent BRJ-related advantage.

**Conclusions:**

Short-term BRJ supplementation was associated with selective rather than generalized recovery benefits after EIMD in trained female volleyball players. The clearest findings were higher CMJ height and calf PPT under BRJ than PLA at 72 h, whereas the remaining outcomes showed no consistent treatment-related differences.

## 1. Introduction

Volleyball places repeated eccentric and concentric demands on the lower limbs within the stretch-shortening cycle, with frequent jumping, landing, deceleration, and rapid changes of direction [1,2]. These actions can contribute to exercise-induced muscle damage (EIMD) and to impaired performance over the next 24–72 h [3]. Recent systematic-review evidence indicates that EIMD can compromise functional performance, including sprint and change-of-direction tasks, across the practical 24–72 h recovery window that matters to team-sport scheduling [4,5]. In female volleyball players, even modest short-term reductions in explosive function may matter because jump performance is closely tied to neuromuscular status, training readiness, and match demands [6,7]. In a jump-dependent team sport, that narrow recovery window can affect both the next training session and short-term match readiness [8].

Beetroot juice (BRJ) is relevant in this setting because it is a practical source of dietary nitrate and has been widely discussed as an ergogenic aid [9,10]. Through the nitrate-nitrite-nitric oxide pathway, BRJ may alter local hemodynamic regulation, contractile behavior, and tolerance to demanding exercise [11–13]. Because EIMD involves secondary inflammation, oxidative stress, microvascular disturbance, and nociceptive sensitization during recovery, increasing nitric oxide bioavailability may provide a physiological basis for using BRJ as a recovery-oriented strategy rather than only as an acute ergogenic aid [3,14–16]. In addition, beetroot contains betalains and other bioactive compounds with potential antioxidant and anti-inflammatory properties, which may further support post-exercise repair processes and fatigue recovery [10,14,15]. What remains less clear is whether these physiological effects translate into broad recovery benefits. Meta-analytic summaries suggest that nitrate-related effects are not uniform and depend on the task, the population, and the supplementation strategy [17,18]. Reviews focused specifically on BRJ and EIMD reach a similar conclusion: some markers of muscle function may improve, but soreness, swelling, and other recovery outcomes do not move consistently in the same direction [14,15]. Recovery after EIMD should therefore not be treated as a single construct [19].

This lack of certainty is especially important in female athletes. Women remain underrepresented in nitrate supplementation research, and sex-related differences in nitrate physiology and exercise responses may influence how any ergogenic or recovery effect is expressed [20,21]. Methodological and sports-nutrition guidance has therefore called for more female-specific evidence in applied settings [22,23]. The existing female BRJ studies illustrate the problem rather than resolving it: acute or short-term supplementation has shown trivial or absent effects on neuromuscular or match-play outcomes in elite female hockey, rugby, and water-polo athletes [24–26]. Under other conditions, BRJ has improved aerobic capacity in female athletes [27]. There is still little evidence from female volleyball players following EIMD, particularly when recovery is assessed across multiple domains rather than with a single performance measure [28].

That gap matters because the outcomes most relevant to applied recovery are not interchangeable. Countermovement jump (CMJ) height is commonly used to track neuromuscular status and short-term readiness in sport [6,29]. A volleyball-related agility task captures a broader movement demand involving braking, re-acceleration, and change-of-direction control [30]. Visual analog scale soreness and pressure pain threshold reflect related but non-identical aspects of pain recovery, whereas thigh circumference provides only a simple field marker of swelling [31,32]. Low-velocity knee isokinetic testing, in turn, isolates a narrower component of muscle function that may not map directly onto explosive sport tasks [33,34]. The unresolved question is therefore not simply whether BRJ aids recovery, but whether any benefit is expressed uniformly across these different outcome domains in female volleyball players [14,28].

The purpose of this study was therefore to examine the effects of short-term BRJ supplementation on recovery from EIMD in trained female volleyball players using a randomized, double-blind, placebo-controlled crossover design. The primary interest was functional recovery, assessed with CMJ height and volleyball-specific agility test time. Pain-related outcomes were treated as secondary markers, and swelling and knee isokinetic outcomes were included to test whether any benefit extended beyond those domains. We expected BRJ to show its clearest effects in functional recovery, with smaller or less consistent effects in the other outcomes.

## 2. Methods

### 2.1. Participants

Seventeen trained female university volleyball players with National Level II athlete certification or higher were recruited. All participants were members of the same university volleyball team and competed in provincial-level university volleyball competitions. The athletes had completed systematic volleyball training for an average of more than six years and engaged in training at least three times per week. However, national-level competition experience was not uniformly distributed across the cohort. Based on the participants’ training history and competitive standard as members of a university volleyball team, the cohort was classified as Tier 2 (Trained/Developmental) according to the Participant Classification Framework [35], which defines this tier as comprising athletes who perform at the local level, train approximately three times per week, and train with the propose of competing. The study was conducted during the off-season. Participants had a mean age of 21 ± 1 years, height of 176 ± 5 cm, and body mass of 65 ± 7 kg. All were free from cardiovascular or metabolic disease and reported no recent musculoskeletal injury. None reported allergy to beetroot or sea buckthorn, and none were taking dietary supplements or medications during the study period. All participants were naturally menstruating women and none reported the use of hormonal contraceptives. Participants were instructed to avoid smoking, alcohol, caffeine, and antibacterial mouthwash because these factors may alter nitrate metabolism. They were also instructed to maintain their habitual diet and the same supervised team training program throughout the study period. The study was approved by the Human Research Ethics Committee of Northeast Normal University (approval number: 20242026) and was conducted in accordance with the Declaration of Helsinki. Written informed consent was obtained before participation. To reduce hormonal confounding, testing was scheduled outside menstruation and, where feasible, during the late follicular phase on the basis of self-reported cycle timing and current recommendations for female exercise research [23,36].

### 2.2. Sample size calculation

Sample size was estimated from the effect reported by Clifford et al. for vertical jump recovery after beetroot juice supplementation following eccentric exercise [37]. Using G*Power 3.1 with a two-tailed test, alpha = 0.05, and power = 0.80, the calculation indicated that at least nine observations per condition were required. This calculation was used as a planning reference for recruitment rather than as a post hoc guarantee of power for every outcome, because the final analysis relied on mixed models after attrition in the second crossover period. Seventeen athletes were recruited to reduce the risk of dropout.

### 2.3. Study design

The study used a randomized, double-blind, placebo-controlled crossover design. Participants first completed a separate familiarization session 48 h before baseline testing to reduce learning effects. During familiarization, all testing procedures were rehearsed, including the knee isokinetic test, the volleyball-specific agility test, the CMJ test, pressure pain threshold assessment, and thigh circumference measurement. After familiarization, baseline assessments were completed. A computer-generated randomization sequence was used to determine treatment order, with nine participants assigned to PLA first and eight to BRJ first. Allocation was implemented by a researcher who retained the beverage code and was not involved in outcome assessment, data processing, or statistical analysis. Participants and outcome assessors remained blinded to treatment allocation, and the code was revealed only after data processing and statistical analyzes had been completed. Each crossover period consisted of baseline testing, the EIMD protocol, and a 72 h recovery window. Baseline testing was completed in the Exercise Science Laboratory of Northeast Normal University. After a standardized 10-min warm-up, participants performed the knee isokinetic test and CMJ test, followed a 15-min rest interval by the volleyball-specific agility test on an indoor court. The EIMD protocol was performed 24 h later. BRJ or placebo was then ingested at 24, 48, and 72 h post-EIMD, and recovery markers were recorded during the same period. This delayed post-EIMD supplementation schedule was selected to focus on the practical recovery phase after the initial muscle-damaging bout, rather than on the acute effects of supplementation immediately after exercise. The 72 h window was chosen because previous BRJ-and-EIMD evidence suggests that recovery effects for isometric strength and countermovement jump performance may emerge across the 24–72 h post-exercise period, while a recent study in female volleyball players monitored recovery only up to 48 h and reported no significant between-condition differences in vertical jump height or volleyball-specific fitness performance [28,37–39]. Functional performance testing was repeated 72 h after the EIMD protocol. A 30-day washout separated the two crossover periods. The overall schedule of testing, supplementation, and crossover is shown in Figure 1.

**Figure 1.**
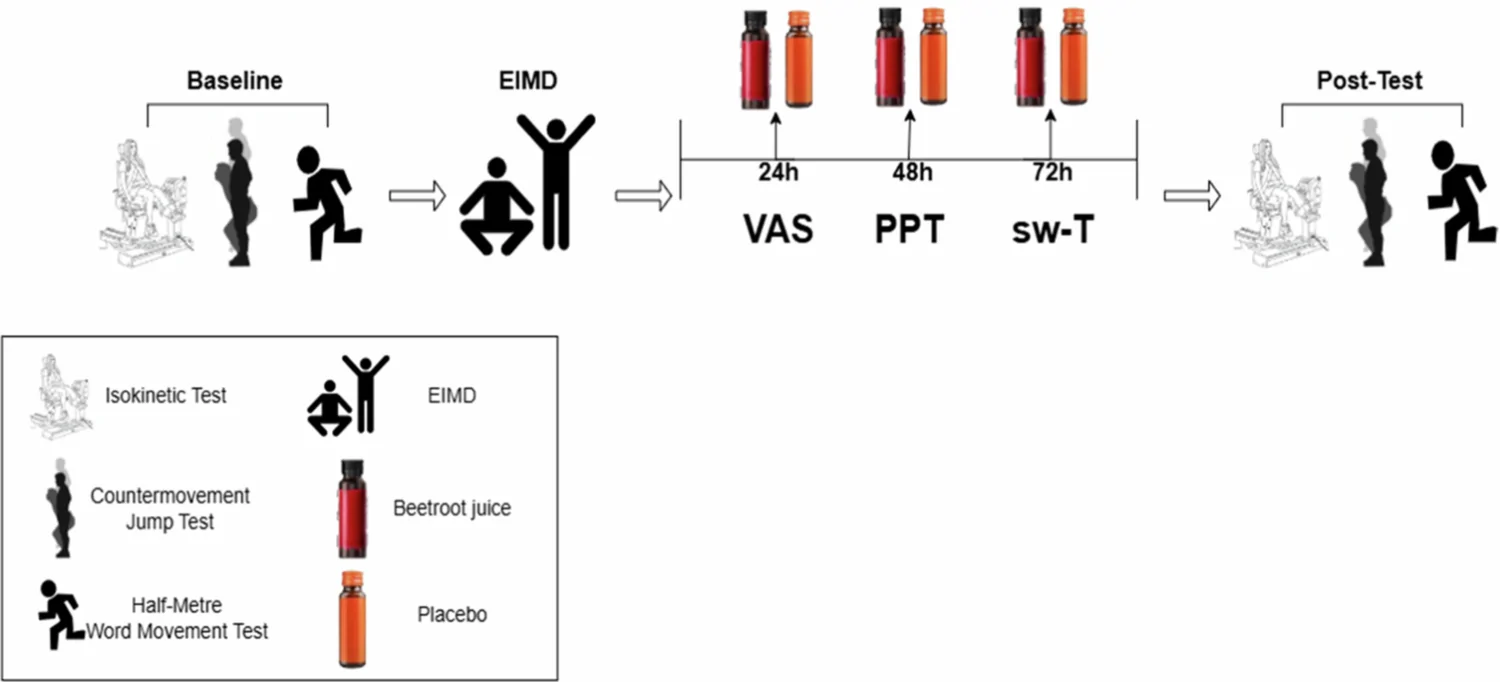
Schematic of the study design. The red and orange bottle icons are schematic labels used only to distinguish the BRJ and comparator conditions; both beverages were administered in identical opaque containers during the trial.

Menstrual cycle status was checked by self-report before each crossover period to avoid testing during menstruation. Before the second crossover period, four participants withdrew because the planned testing dates overlapped with menstruation in accordance with the predefined exclusion criteria. As a result, the second period included five participants in PLA and eight in BRJ. This imbalance was handled in the primary mixed-model analysis and in supplementary robustness checks described below.

### 2.4. Exercise-induced muscle damage protocol (EIMD)

The EIMD protocol was designed around repeated loaded vertical jumps. After a 10-min warm-up on a bicycle ergometer, participants performed 200 maximal weighted vertical jumps while wearing a vest corresponding to 10% of body mass. The protocol consisted of 10 sets of 20 jumps separated by 2 min of passive rest. This loading model was chosen because it aligns with previous BRJ-and-EIMD protocols [37] and with recent work using a comparable loaded-jump protocol in female volleyball players [28].

### 2.5. Supplementation procedures

In the BRJ condition, participants ingested 50 mL of concentrated beetroot juice (Maxwin, China), providing an estimated 5 mmol (approximately 310 mg) of nitrate per serving on the basis of manufacturer information. This per-serving dose lies near the lower end of the range examined in primary dose-response research [13], consistent with the lower range summarized in recent meta-analytic evidence [18]. Previous team-sport research has shown that nitrate-rich BRJ ingestion can substantially increase plasma nitrite concentration compared with a low-nitrate placebo [40]. In the PLA condition, participants ingested 50 mL of sea buckthorn concentrate (Mengzhiqi, China). According to the manufacturer's ingredient list, the product contained sea buckthorn concentrate only, with no other additives. Both beverages were administered in identical opaque containers, and the comparison beverage was selected to preserve blinding. Although sea buckthorn contains polyphenols and other phytochemicals, evidence supporting fruit-derived polyphenols for post-EIMD recovery generally involves targeted, high-dose, multi-day supplementation, which differs from the low-volume comparator beverage used in the present study [41]. The estimated nutritional composition of the BRJ and PLA beverages, obtained from manufacturer-provided product information, is presented in Table 1; the study batches were not independently analyzed. Supplementation was administered under supervision at 24, 48, and 72 h post-EIMD. At each time point, the recovery-marker assessment sequence, including VAS, PPT, and thigh circumference, commenced immediately after beverage ingestion. At 72 h, functional performance and knee isokinetic testing commenced 2 h after ingestion. Participants remained blinded to treatment allocation. No dose was administered immediately after the EIMD protocol; therefore, the study evaluated a delayed recovery-phase supplementation strategy. Based on the manufacturer-stated nitrate content and measured body mass, the estimated relative nitrate dose was 4.8 ± 0.5 mg·kg−1 per serving (range 4.1–5.5 mg·kg−1).

**Table 1.** Nutritional composition of the BRJ and placebo beverages (estimated).

	Amount per 50 mL	
Details	BRJ	PLA
Energy (kJ)	145	111
Carbohydrates (g)	7.5	5.7
Fat (g)	0	0
Protein (g)	1	0.3
Na⁺ (mg)	80	40
Nitrate (mmol)	5	<0.5

Note: Values were estimated from manufacturer-provided product information. The study batches were not independently analyzed for nitrate or other constituents.

### 2.6. Recovery markers

Delayed-onset muscle soreness was assessed with a standardized 10-cm visual analog scale (VAS). The left end of the scale indicated no pain and the right end indicated maximum pain. Participants marked the line according to perceived lower-limb soreness, and the distance from the left endpoint to the mark was measured to the nearest 1 mm. VAS was assessed at 24, 48, and 72 h post-EIMD, in line with procedures used in team-sport recovery research [28,42].

Thigh circumference was used as a simple swelling marker. With participants standing upright, the greater trochanter and tibial tuberosity were identified and marked. Circumference was then measured three times with a flexible tape to the nearest 1 mm while keeping the tape horizontal and avoiding compression of the skin. The mean of the three measurements was retained. This field-based approach is consistent with previous BRJ-and-EIMD studies that included simple swelling assessment [28,37].

Pressure pain threshold (PPT) was operationally defined as the cuff pressure at which the participant first reported pain at the predefined thigh and calf sites. Measurements were performed using a calibrated manual pneumatic cuff system (Theratools, TZ-brf-0347). Participants were seated, and the cuff was positioned at the midpoint of the target region. The same investigator increased cuff pressure at approximately 20 mmHg/s until the participant first reported pain. Two measurements were obtained at each site and time point, and the mean value was used for analysis. The same operational procedure was used at 24, 48, and 72 h post-EIMD. Because the endpoint was the first onset of pain rather than the maximum tolerable pressure, the measure represented a pressure pain threshold rather than pressure pain tolerance [28,43,44].

### 2.7. Tests functional performance and knee isokinetic testing

Knee extensor and flexor strength was assessed with an isokinetic dynamometer (CONN-TREX-MJ, PHYSIOMED, Germany). Participants performed five maximal consecutive concentric repetitions at an angular velocity of 60°/s. The chair back was set at 85 degrees, and the torso, thigh, and tibia were stabilized with straps to minimize extraneous movement. The dynamometer axis was aligned with the lateral femoral epicondyle.

For the countermovement jump (CMJ), participants stood on a force platform with their hands on their hips, performed a rapid downward movement, and immediately executed a maximal vertical jump. Three trials were completed with 1 min of rest between trials, and mean jump height was calculated from flight time. CMJ was included because it is a sensitive field-based indicator of neuromuscular status and is widely used in BRJ and EIMD research [6,37].

The volleyball-specific agility test was used to assess sport-specific movement performance. Participants started the timing at point O, completed the predetermined movement sequence shown in Figure 2, and returned to point O to stop the timer. If the sensor bottle was not activated successfully, the attempt was repeated. Two trials were performed and the best time was recorded. The test layout was adapted from a recent volleyball study that included agility assessment [45].

**Figure 2.**
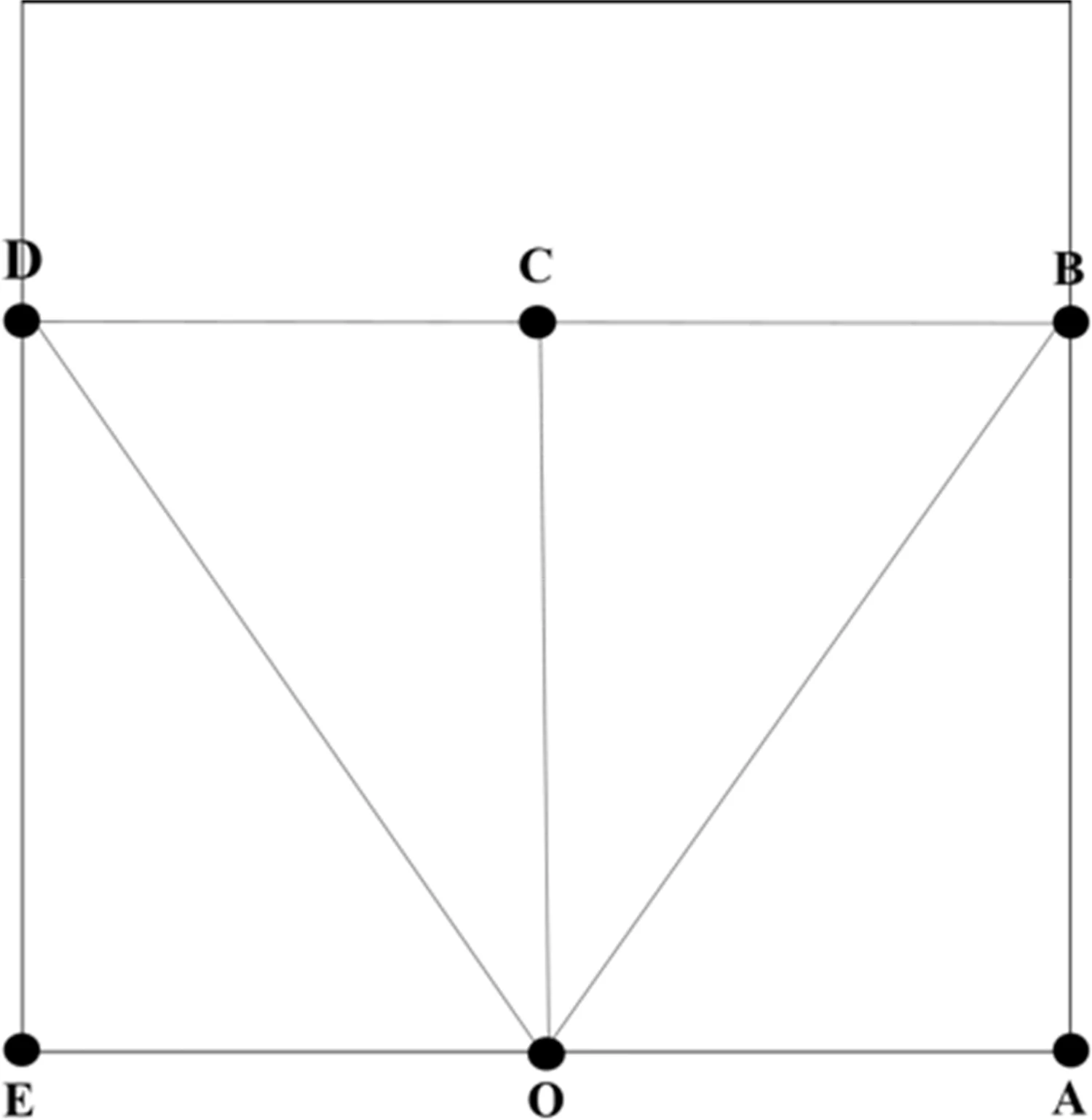
Schematic diagram of the volleyball-specific agility test.

### 2.8. Statistical analysis

All analyzes were performed in R (version 4.5.3). Seventeen participants were randomized, but four did not complete the second crossover period. To preserve the paired crossover structure, the primary inferential analysis was performed in the 13 athletes who completed both periods. The main inferential emphasis was placed on the two functional outcomes specified in the Introduction: CMJ height and volleyball-specific agility test time. Pain-related outcomes (VAS, thigh PPT, and calf PPT) were interpreted as secondary markers, whereas thigh circumference and knee isokinetic variables were treated as supportive extension outcomes.

Linear mixed models were used for all inferential analyzes. For CMJ height, volleyball-specific agility test time, visual analog scale soreness, thigh pressure pain threshold, calf pressure pain threshold, and thigh circumference, each model included treatment condition (BRJ vs PLA), time, crossover period (period 1 vs period 2), and sequence (BRJ-first vs PLA-first) as fixed effects, with participant entered as a random intercept. For bilateral knee isokinetic outcomes (peak torque, mean power, peak power, and time to peak torque), side and relevant interaction terms were added to the fixed-effects structure.

Immediate outcomes were modeled using baseline and 72 h post-EIMD values. Recovery outcomes were modeled using the available post-EIMD time points (24, 48, and 72 h). Baseline thigh circumference was retained descriptively because it was available in the source files; however, the recovery model for this variable focused on the repeated post-EIMD observations.

Model-based F tests were obtained from the fitted mixed models. Planned post hoc contrasts were estimated within the same mixed-model framework and adjusted for multiplicity using the Holm procedure within each outcome-specific contrast family. For immediate outcomes, these contrasts included between-condition comparisons at baseline and 72 h post-EIMD, together with within-condition baseline-versus-72 h contrasts. For recovery outcomes, BRJ-versus-PLA contrasts were tested at 24, 48, and 72 h post-EIMD, and within-condition recovery contrasts were tested for 24 h versus 48 h, 24 h versus 72 h, and 48 h versus 72 h.

Standardized effect sizes for post hoc comparisons were expressed as model-based Cohen's d, calculated as the estimated contrast divided by the residual standard deviation of the corresponding mixed model. Approximate partial eta-squared values were also calculated for fixed-effect tests to provide descriptive effect-size information. Effect sizes were interpreted as trivial (< 0.20), small (0.20–0.49), moderate (0.50–0.79), or large (≥ 0.80). Descriptive data are presented as mean ± SD, and statistical significance was set at *P* < 0.05.

Crossover-period-specific descriptive statistics for VAS and thigh pressure pain threshold were summarized separately because these outcomes were assessed only during the post-EIMD recovery window. As an additional robustness check, a supplementary crossover period 1-only sensitivity analysis was performed for the key functional and recovery outcomes to illustrate how the main contrasts behaved in the smaller single-period subset.

## 3. Results

### 3.1. Functional performance outcomes

Among the prespecified functional outcomes, CMJ remained the clearest signal in the corrected completers-only dataset (Figure 3; Table 2). The mixed model showed significant main effects of treatment (*P* = 0.025) and time (*P* < 0.001), whereas the treatment × time interaction showed a trend toward statistical significance (*P* = 0.062; Supplementary Table S1). Planned post hoc contrasts showed that CMJ height was greater under BRJ than PLA at 72 h post-EIMD (mean difference 1.5 cm, 95% CI 0.05 to 2.97, adjusted *P* = 0.033, d = 1.01; Supplementary Table S2). Within-condition contrasts showed a significant decline from baseline to 72 h under PLA (mean change −2.5 cm, adjusted *P* < 0.001, d = −1.67), whereas the corresponding change under BRJ was not significant (mean change −0.9 cm, adjusted *P* = 0.127, d = −0.60; Supplementary Table S4). Descriptive CMJ values were 36.1 ± 4.5 cm at baseline and 33.6 ± 4.0 cm at 72 h under PLA, and 36.3 ± 3.3 cm and 35.4 ± 3.4 cm, respectively, under BRJ (Table 2).

**Table 2.** Descriptive statistics for volleyball-specific agility test time, countermovement jump (CMJ) height, and knee isokinetic test variables measured at baseline and 72 h post-EIMD under placebo (PLA) and beetroot juice (BRJ) conditions.

Domain	Outcome	Side	PLA baseline	PLA 72 h post-EIMD	BRJ baseline	BRJ 72 h post-EIMD
Functional performance	CMJ height (cm)		36.1 ± 4.5	33.6 ± 4.0	36.3 ± 3.3	35.4 ± 3.4
Functional performance	Volleyball-specific agility test time (s)		17.7 ± 0.8	18.1 ± 0.9	17.8 ± 0.9	18.0 ± 0.9
Knee isokinetic test	Peak torque (Nm)	Left	45.2 ± 5.8	45.7 ± 7.1	42.4 ± 6.6	44.3 ± 5.3
Knee isokinetic test	Peak torque (Nm)	Right	46.9 ± 7.9	48.2 ± 7.5	44.9 ± 5.9	45.7 ± 7.3
Knee isokinetic test	Mean power (W)	Left	55.7 ± 7.5	55.3 ± 6.6	54.2 ± 6.7	52.8 ± 5.9
Knee isokinetic test	Mean power (W)	Right	56.1 ± 7.6	58.3 ± 5.8	57.3 ± 4.5	55.3 ± 9.0
Knee isokinetic test	Peak power (W)	Left	61.2 ± 32.1	59.2 ± 29.1	68.5 ± 28.9	65.6 ± 24.0
Knee isokinetic test	Peak power (W)	Right	97.2 ± 12.6	98.1 ± 13.3	96.4 ± 9.6	92.2 ± 14.7
Knee isokinetic test	Time to peak torque (ms)	Left	43.5 ± 13.0	38.1 ± 11.4	40.8 ± 11.2	35.5 ± 9.8
Knee isokinetic test	Time to peak torque (ms)	Right	42.8 ± 9.1	48.6 ± 13.3	45.1 ± 15.6	47.0 ± 19.5

Note: Values are mean ± SD. Baseline and 72 h post-EIMD values were pooled across both crossover periods by treatment condition (PLA, *n *= 14 observations; BRJ, *n *= 16 observations). Knee isokinetic outcomes were assessed at 60°/s and are shown by anatomical side (left and right); leg dominance was not recorded. Detailed fixed-effect tests are provided in Supplementary Table S1, planned post hoc contrasts in Supplementary Table S2, and within-condition baseline-to-72 h changes in Supplementary Table S4.

**Figure 3.**
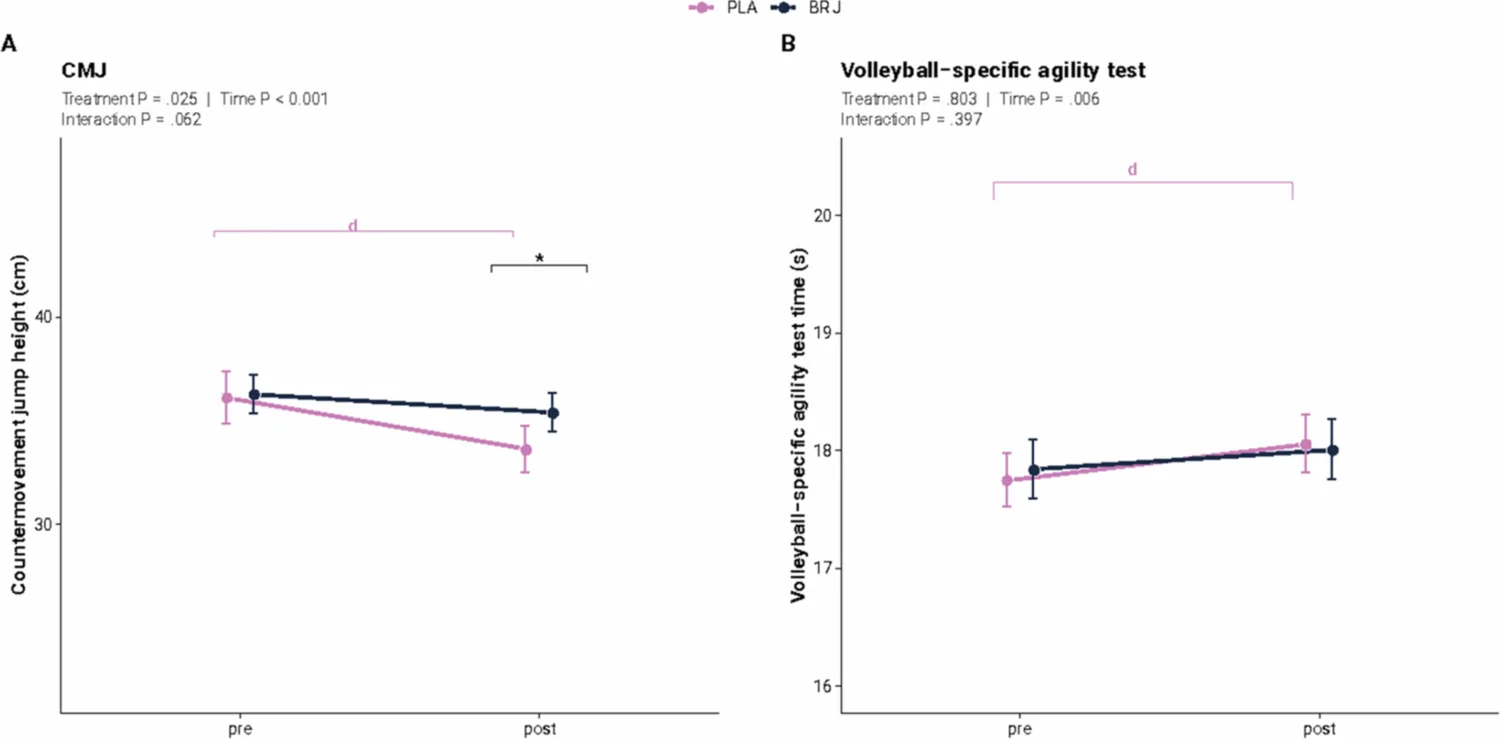
Functional performance outcomes under placebo (PLA) and beetroot juice (BRJ) conditions. Panel A shows countermovement jump (CMJ) height (cm), and panel B shows volleyball-specific agility test time (s), measured at baseline and 72 h post-exercise-induced muscle damage (EIMD). Individual observations are overlaid on treatment means with standard errors. Treatment, time, and treatment × time *P* values are from linear mixed models. Black asterisks denote Holm-adjusted between-condition planned post hoc comparisons at the same time point (*P < 0.05, **P < 0.01, ***P < 0.001). Colored lowercase (d) denotes a significant within-condition baseline-versus-72 h post-EIMD comparison, with bracket color matching the treatment condition.

The volleyball-specific agility test did not show a clear treatment-related effect. The mixed model identified a main effect of time (*P* = 0.006), whereas neither the treatment effect nor the treatment × time interaction was significant (*P* = 0.803 and *P* = 0.397, respectively; Supplementary Table S1). Within-condition contrasts showed that PLA performance worsened from baseline to 72 h post-EIMD (mean change 0.3 s, adjusted *P* = 0.029, d = 1.05), whereas the corresponding change under BRJ was not significant (mean change 0.2 s, adjusted *P* = 0.420, d = 0.58; Supplementary Table S4). Descriptively, agility time increased from 17.7 ± 0.8 s to 18.1 ± 0.9 s under PLA, but changed less under BRJ, from 17.8 ± 0.9 s to 18.0 ± 0.9 s (Table 2).

### 3.2. Recovery markers

Among the secondary recovery markers, calf pressure pain threshold was the clearest treatment-related signal (Figure 4; Table 3). The mixed model showed a significant treatment × time interaction (*P* < 0.001), whereas neither the treatment main effect (*P* = 0.104) nor the time main effect (*P* = 0.123) was significant (Supplementary Table S1). Planned post hoc contrasts showed that calf PPT was higher under BRJ than PLA at 72 h post-EIMD (mean difference 47.5 mmHg, 95% CI 11.80 to 83.28, adjusted *P* = 0.004, d = 1.26; Supplementary Table S2). Within-condition contrasts further showed that calf PPT increased from 24 h to 48 h (mean change 49.3 mmHg, adjusted *P* = 0.004, d = 1.30) and from 24 h to 72 h (mean change 58.9 mmHg, adjusted *P* < 0.001, d = 1.56) under BRJ, whereas no corresponding within-condition changes were significant under PLA (Supplementary Table S4). Descriptively, calf PPT increased from 182.2 ± 45.2 mmHg at 24 h to 241.1 ± 28.0 mmHg at 72 h under BRJ, whereas the comparable PLA values were 215.0 ± 46.3 and 192.8 ± 43.9 mmHg, respectively (Table 3). The 72 h calf PPT contrast also remained directionally stable in the crossover period 1-only sensitivity analysis (Supplementary Table S5).

**Table 3.** Descriptive statistics for recovery markers measured before and during the 72 h recovery period after EIMD under placebo (PLA) and beetroot juice (BRJ) conditions.

Outcome	PLA baseline	PLA 24 h	PLA 48 h	PLA 72 h	BRJ baseline	BRJ 24 h	BRJ 48 h	BRJ 72 h
Thigh pressure pain threshold (mmHg)		163.9 ± 43.9	147.2 ± 36.5	159.2 ± 37.2		152.5 ± 30.3	156.0 ± 26.6	169.5 ± 36.6
Calf pressure pain threshold (mmHg)		215.0 ± 46.3	204.6 ± 48.2	192.8 ± 43.9		182.2 ± 45.2	231.5 ± 34.4	241.1 ± 28.0
Thigh circumference (cm)	53.9 ± 2.7	54.1 ± 2.7	54.2 ± 2.6	54.3 ± 2.6	54.4 ± 3.4	54.3 ± 3.4	54.5 ± 3.4	54.4 ± 3.4
VAS score (mm)		37.8 ± 10.7	33.2 ± 10.7	26.3 ± 11.7		41.9 ± 8.2	35.3 ± 7.5	23.7 ± 7.0

Note: Values are mean ± SD. Baseline values were available only for thigh circumference; VAS and pressure pain threshold outcomes were assessed at 24, 48, and 72 h post-EIMD. Descriptive statistics were pooled across both crossover periods by treatment condition (PLA, *n *= 14 observations; BRJ, *n *= 16 observations). Detailed fixed-effect tests are provided in Supplementary Table S1, planned between-condition contrasts in Supplementary Table S2, crossover-period-specific VAS and thigh PPT descriptives in Supplementary Table S3, and within-condition recovery changes in Supplementary Table S4.

**Figure 4.**
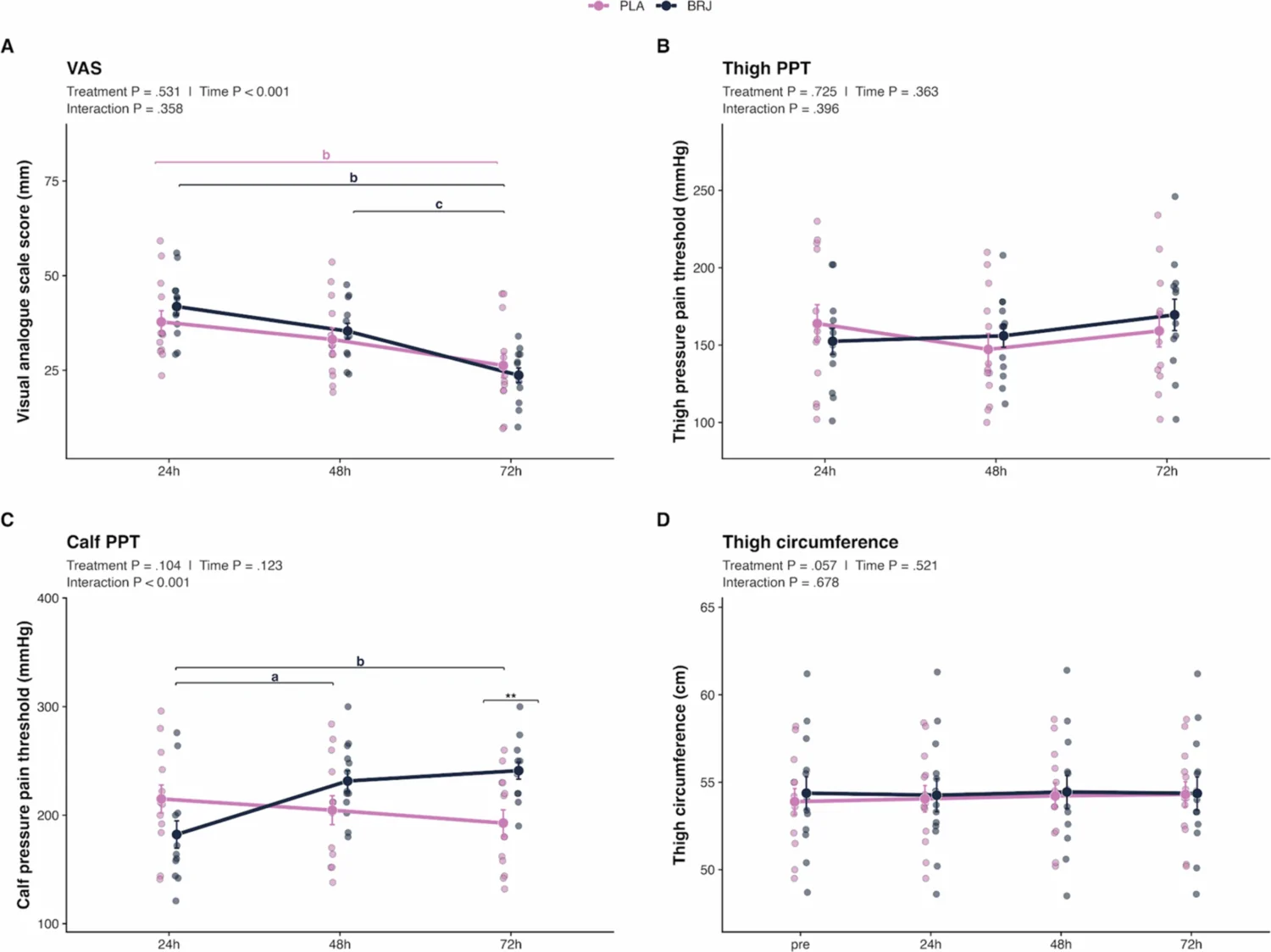
Recovery markers under placebo (PLA) and beetroot juice (BRJ) conditions. Panel A shows visual analog scale (VAS) soreness (mm), panel B shows thigh pressure pain threshold (mmHg), panel C shows calf pressure pain threshold (mmHg), and panel D shows thigh circumference (cm). VAS and pressure pain threshold outcomes were assessed at 24, 48, and 72 h post-EIMD. Thigh circumference is shown at baseline, 24, 48, and 72 h, with baseline retained descriptively. Individual observations are overlaid on treatment means with standard errors. Treatment, time, and treatment × time *P* values are from linear mixed models. Black asterisks, when present, denote Holm-adjusted between-condition planned post hoc comparisons at matched time points (*P* < 0.05, *P* < 0.01, *P* < 0.001). Colored lowercase letters, when present, denote significant within-condition comparisons for 24 h vs 48 h (a), 24 h vs 72 h (b), and 48 h vs 72 h (c); bracket color matches the treatment condition.

VAS changed over time but did not show a clear treatment-related difference. The mixed model showed a strong main effect of time (*P* < 0.001), with no treatment effect (*P* = 0.531) and no treatment × time interaction (*P* = 0.358; Supplementary Table S1). None of the between-condition contrasts at 24, 48, or 72 h remained significant after Holm adjustment (Supplementary Table S2). Within-condition contrasts showed that VAS declined from 24 h to 72 h under both PLA (mean change −11.5 mm, adjusted *P* = 0.003, d = −1.35) and BRJ (mean change −18.2 mm, adjusted *P* < 0.001, d = −2.12). In addition, VAS declined from 48 h to 72 h under BRJ (mean change −11.7 mm, adjusted *P* = 0.003, d = −1.36; Supplementary Table S4). Descriptively, VAS decreased from 37.8 ± 10.7 mm to 26.3 ± 11.7 mm under PLA and from 41.9 ± 8.2 mm to 23.7 ± 7.0 mm under BRJ (Table 3). Crossover-period-specific descriptive values are provided in Supplementary Table S3.

Thigh pressure pain threshold showed no clear BRJ-related effect. The mixed model showed no significant treatment effect, time effect, or treatment × time interaction (*P* = 0.725, *P* = 0.363, and *P* = 0.396, respectively; Supplementary Table S1). None of the planned BRJ-versus-PLA contrasts at 24, 48, or 72 h remained significant after multiplicity adjustment (Supplementary Table S2), and no within-condition recovery contrast survived Holm adjustment (Supplementary Table S4). At 72 h, mean ± SD values were 169.5 ± 36.6 mmHg under BRJ and 159.2 ± 37.2 mmHg under PLA (Table 3); the corresponding contrast did not reach significance (mean difference 12.1 mmHg, adjusted *P* = 1.000; Supplementary Table S2). Because this outcome showed some between-period variability, crossover-period-specific descriptive statistics are presented separately in Supplementary Table S3.

Thigh circumference did not show a clear treatment-related recovery pattern. The mixed model showed a small treatment trend (*P* = 0.057), but no time effect (*P* = 0.521) and no treatment × time interaction (*P* = 0.678; Supplementary Table S1). Planned between-condition contrasts at baseline, 24 h, 48 h, and 72 h were all non-significant, and no within-condition change contrast survived adjustment (Supplementary Tables S2 and S4). Descriptively, thigh circumference remained broadly similar between conditions across the recovery period (Table 3).

### 3.3. Knee isokinetic test outcomes

None of the low-velocity knee isokinetic outcomes showed a consistent BRJ-related difference (Figure 5; Table 2). Peak torque showed significant main effects of treatment (*P* = 0.011), side (*P* = 0.019), and period (*P* = 0.040), but no significant treatment × time interaction or higher-order interaction involving side (Supplementary Table S1). Mean power showed a side effect (*P* = 0.036), but no significant treatment, time, or treatment × time effects. Peak power showed strong side and period effects (both *P* < 0.001), whereas time to peak torque showed a side effect (*P* = 0.003) and a time × side interaction (*P* = 0.033; Supplementary Table S1).

**Figure 5.**
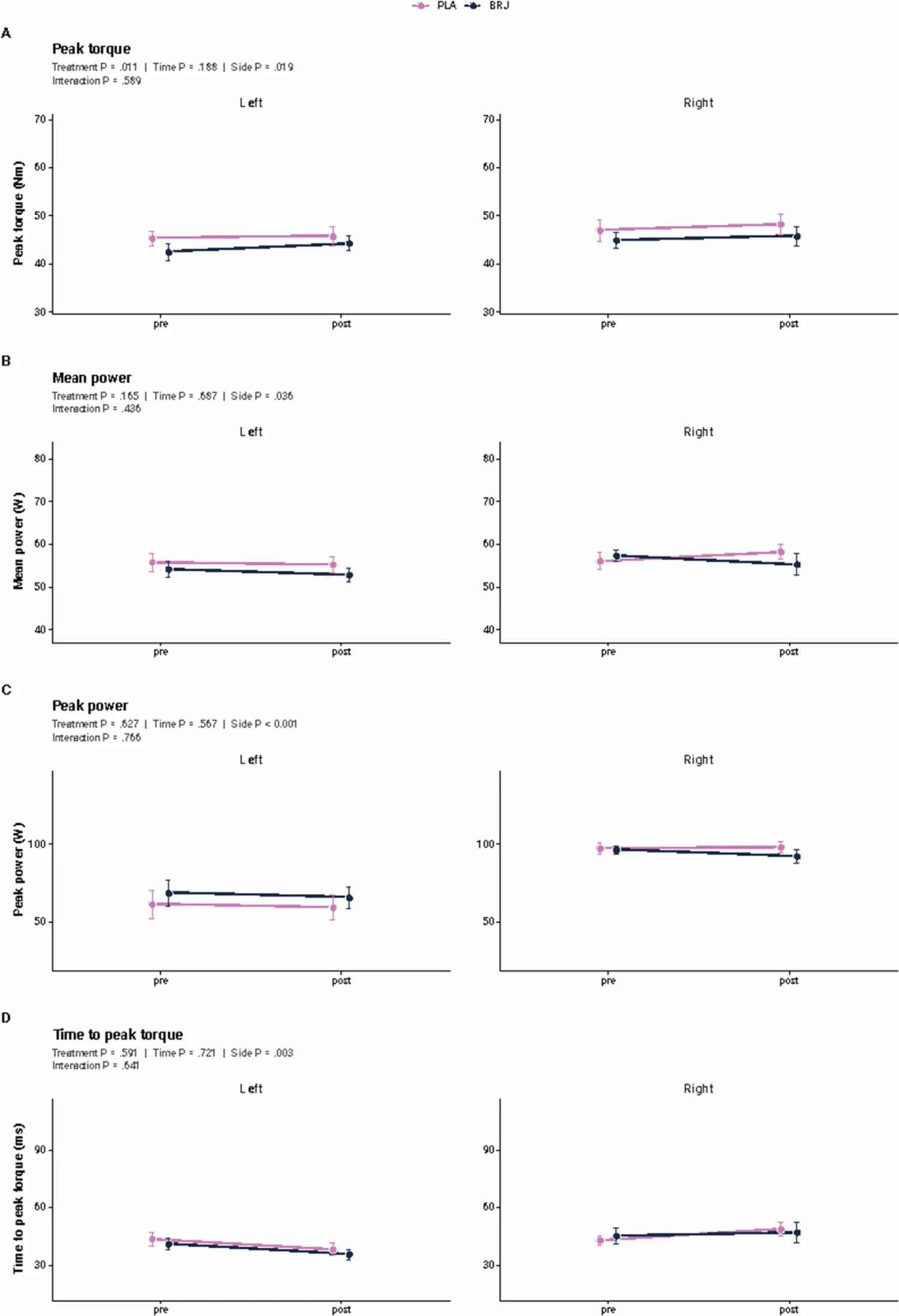
Knee isokinetic test outcomes under placebo (PLA) and beetroot juice (BRJ) conditions. Panel A shows peak torque (Nm), panel B shows mean power (W), panel C shows peak power (W), and panel D shows time to peak torque (ms), measured during knee isokinetic testing at 60°/s. Left and right limbs are shown in separate facets. Individual observations are overlaid on treatment means with standard errors. Treatment, time, side, and relevant interaction *P* values are from linear mixed models. Black asterisks denote Holm-adjusted between-condition planned post hoc comparisons at the same time point (*P < 0.05, **P < 0.01, ***P < 0.001). Colored lowercase (d) denotes a significant within-condition baseline-versus-72 h post-EIMD comparison, with bracket color matching the treatment condition.

Despite these model-level effects, none of the planned BRJ-versus-PLA contrasts at baseline or 72 h post-EIMD was significant for any knee isokinetic outcome, and no within-condition baseline-to-72 h contrast survived multiplicity adjustment (Supplementary Tables S2 and S4). These findings indicate that the corrected completers-only analysis did not show a consistent BRJ-related advantage for low-velocity isolated knee muscle function.

## 4. Discussion

Taken together, the present data support a selective rather than generalized recovery pattern after short-term BRJ supplementation in trained female volleyball players following EIMD. The clearest treatment-related finding was a higher CMJ height under BRJ than PLA at 72 h post-EIMD. Calf pressure pain threshold provided a secondary, more localized finding, whereas agility, VAS, thigh PPT, thigh circumference, and low-velocity knee isokinetic outcomes did not show comparably clear treatment-related effects. The sensitivity analyzes were less precise and were not interpreted as confirmatory. Importantly, although the 72 h CMJ contrast favored BRJ, the overall treatment × time interaction showed a trend toward statistical significance (*P* = 0.062) but did not reach the prespecified threshold; therefore, the 72 h contrast should be interpreted as a time-point-specific difference rather than as evidence that BRJ altered the entire recovery trajectory.

That pattern is easier to understand when the outcomes are read hierarchically rather than as interchangeable markers of "recovery." Among the prespecified functional outcomes, CMJ carried the clearest evidential weight. CMJ is widely used to track neuromuscular status and short-term recovery in athletes [6,29]. In the corrected analysis, CMJ declined significantly from baseline to 72 h under PLA, whereas the corresponding change under BRJ was not significant; CMJ was also higher under BRJ than PLA at 72 h post-EIMD. That result is closely aligned with the eccentric-exercise study by Clifford et al., who also reported faster restoration of jump-related function after BRJ supplementation [37]. However, because the treatment × time interaction did not reach significance, this result is more appropriately interpreted as a late between-condition difference in CMJ than as definitive evidence that BRJ altered the entire recovery trajectory. Under PLA, mean CMJ height decreased by 2.5 cm from baseline to 72 h (approximately 6.9%). This suggests that the protocol elicited a moderate functional decrement rather than severe impairment, which may partly explain the absence of clear treatment-related differences in thigh circumference, VAS soreness, and low-velocity isokinetic outcomes. By contrast, the volleyball-specific agility test showed only a significant effect of time, with no treatment effect or treatment × time interaction. The direct BRJ-versus-PLA contrasts did not support a clear treatment-related advantage. That difference is plausible because CMJ is a constrained explosive task, whereas agility testing is more composite and depends on braking, re-acceleration, movement strategy, and change-of-direction control [30]. Athlete-monitoring studies make a similar point: simple explosive tests often change more clearly than technically richer change-of-direction outcomes [29,46]. Recent evidence also indicates that EIMD can impair sprint and change-of-direction performance across the 24–72 h recovery window, but the magnitude and detectability of impairment vary across functional tests [5]. Taken together, the functional data fit better with a task-sensitive interpretation than with a broad claim that BRJ improves recovery across every performance outcome.

The same selective reading helps interpret the pain-related outcomes. Calf PPT was the only secondary recovery marker to show a clear between-condition difference at 72 h, whereas VAS and thigh PPT did not. In addition, calf PPT increased from 24 h to 48 h and from 24 h to 72 h under BRJ, whereas no corresponding within-condition recovery contrast was significant under PLA. This resembles the delayed protection reported by Clifford and colleagues after eccentric exercise [37] and is consistent with broader DOMS literature showing that soreness ratings, localized mechanical sensitivity, and functional recovery do not necessarily move in parallel after muscle-damaging exercise [19,31,47]. Recent evidence further suggests that DOMS may selectively alter mechanical sensitivity rather than all pain-related processes in parallel [31]. In that context, calf PPT is better interpreted as a higher cuff-based pressure pain threshold at 72 h than as evidence of generalized analgesia. VAS and thigh PPT did not show a consistent treatment-related pattern.

The null findings outside those domains also help define the boundary of the result. Thigh circumference did not show a clinically interpretable treatment-related pattern, and the low-velocity knee isokinetic outcomes did not show a consistent BRJ advantage. This remained the case despite several isolated model-level effects involving treatment, side, or period. Those outcomes sample different parts of the recovery process: thigh circumference is a coarse field marker of swelling, and low-velocity dynamometer testing isolates a narrower component of muscle function than explosive sport tasks [32,33,48]. Recent BRJ literature makes a similar point. Any benefit appears more likely when the outcome depends on rapid force production or explosive function than when it depends on slower, more isolated muscle actions [33,34,37,39,49–51]. A systematic review of beetroot supplementation after EIMD similarly concluded that the most consistent benefits were observed for functional recovery and muscle soreness, whereas markers of muscle damage, inflammation, and oxidative stress were less consistently responsive [15]. The present dataset therefore supports a narrower practical reading: BRJ may help preserve selected explosive and localized recovery features, but it did not produce a broad across-the-board recovery advantage.

A mechanistic explanation is possible, but it should remain modest. Nitrate-derived nitric oxide may influence local hemodynamic regulation and some aspects of contractile or nociceptive function [11,52], and beetroot juice also contains other phytochemicals that may contribute to recovery responses [10,51,53]. At the same time, the present design does not allow a clean mechanistic attribution to nitrate alone. The sea buckthorn comparator was provided in identical opaque containers to support blinding, but it was not chemically matched and cannot be assumed to have been biologically inert because its phytochemical content was not quantified [54]. Its bioactive constituents may have affected recovery or interacted with BRJ; therefore, the present study cannot isolate nitrate-specific effects. Future studies should use nitrate-depleted beetroot juice or a chemically matched synthetic comparator. The female-athlete context still adds practical value because female team-sport athletes remain underrepresented in this literature [20,22,23], but the main contribution of the study is not a sex-specific mechanism claim. It is the more limited observation that, in trained female volleyball players, any BRJ-related benefit was unevenly distributed across the recovery profile.

These strengths and limits should be read together. The study used a randomized, double-blind, placebo-controlled crossover design, an EIMD model, and a multidimensional recovery assessment. The main limits are the modest sample size, attrition in the second crossover period, the resulting reliance on a corrected completers-only primary analysis, the absence of plasma nitrate or nitrite measurements and batch-specific verification of beverage nitrate content, the absence of CK, LDH, CRP, and other biochemical markers needed to directly verify muscle damage or inflammation, the field-based nature of the PPT procedure, the absence of recorded leg-dominance data, the restriction of knee isokinetic testing to 60°/s, and the absence of hormonal assays despite attempts to reduce menstrual-cycle-related confounding. Accordingly, the observed post-exercise response should be interpreted as functional decrements and localized soreness consistent with EIMD rather than as direct quantification of structural muscle damage or inflammation. The 72 h follow-up window may also have missed later recovery responses. Overall, the clearest findings were a higher CMJ value and a higher calf PPT under BRJ than PLA at 72 h post-EIMD, without consistent treatment-related differences across the remaining recovery outcomes. Future work should test whether that pattern persists in larger female cohorts, in repeated-match or repeated-training models, and alongside direct measures of nitrate exposure, blood flow, muscle damage, and more standardized pain-sensitivity assessment.

## Supplementary Material

Supplementary Material Supplementary_Table_S5_sensitivity_analyses.docx

Supplementary Material Supplementary_Table_S4_within_condition_change_effect_sizes.docx

Supplementary Material Supplementary_Table_S3_phase_specific_VAS_ThighPPT.docx

Supplementary Material Supplementary_Table_S2_planned_posthoc_contrasts.docx

Supplementary Material Supplementary_Table_S1_fixed_effect_tests.docx
