# Supplementary material for: Selective recovery effects of short-term beetroot juice supplementation after exercise-induced muscle damage in trained female volleyball players
Source: J Int Soc Sports Nutr. 2026 Oct 20;23(1):2751785. doi: 10.1080/15502783.2026.2751785 (PMC13686210; doi:10.1080/15502783.2026.2751785)
Supplement: Supplementary file 1 [file RSSN_A_2751785_SM8373.docx]

**Supplementary_Table_S1_fixed_effect_tests**

**Supplementary Table S1. Fixed-effect tests from linear mixed models.**

| **Outcome** | **Effect** | **Num df** | **Den df** | **F** | **P** |
| --- | --- | --- | --- | --- | --- |
| Countermovement jump (CMJ) height (cm) | Treatment | 1 | 35 | 5.50 | 0.025 |
| Countermovement jump (CMJ) height (cm) | Time | 1 | 35 | 16.68 | <0.001 |
| Countermovement jump (CMJ) height (cm) | Period | 1 | 35 | 7.10 | 0.012 |
| Countermovement jump (CMJ) height (cm) | Sequence | 1 | 11 | 0.12 | 0.741 |
| Countermovement jump (CMJ) height (cm) | Treatment Time | 1 | 35 | 3.71 | 0.062 |
| Volleyball-specific agility test time (s) | Treatment | 1 | 35 | 0.06 | 0.803 |
| Volleyball-specific agility test time (s) | Time | 1 | 35 | 8.67 | 0.006 |
| Volleyball-specific agility test time (s) | Period | 1 | 35 | 0.05 | 0.819 |
| Volleyball-specific agility test time (s) | Sequence | 1 | 11 | 0.03 | 0.865 |
| Volleyball-specific agility test time (s) | Treatment Time | 1 | 35 | 0.74 | 0.397 |
| Peak torque (Nm) | Treatment | 1 | 83 | 6.80 | 0.011 |
| Peak torque (Nm) | Time | 1 | 83 | 1.76 | 0.188 |
| Peak torque (Nm) | Side | 1 | 83 | 5.72 | 0.019 |
| Peak torque (Nm) | Period | 1 | 83 | 4.37 | 0.040 |
| Peak torque (Nm) | Sequence | 1 | 11 | 1.41 | 0.260 |
| Peak torque (Nm) | Treatment Time | 1 | 83 | 0.07 | 0.798 |
| Peak torque (Nm) | Treatment Side | 1 | 83 | 0.01 | 0.928 |
| Peak torque (Nm) | Time Side | 1 | 83 | 0.00 | 0.964 |
| Peak torque (Nm) | Treatment Time Side | 1 | 83 | 0.29 | 0.589 |
| Mean power (W) | Treatment | 1 | 83 | 1.96 | 0.165 |
| Mean power (W) | Time | 1 | 83 | 0.16 | 0.687 |
| Mean power (W) | Side | 1 | 83 | 4.53 | 0.036 |
| Mean power (W) | Period | 1 | 83 | 0.34 | 0.562 |
| Mean power (W) | Sequence | 1 | 11 | 3.40 | 0.092 |
| Mean power (W) | Treatment Time | 1 | 83 | 1.48 | 0.227 |
| Mean power (W) | Treatment Side | 1 | 83 | 0.29 | 0.594 |
| Mean power (W) | Time Side | 1 | 83 | 0.22 | 0.642 |
| Mean power (W) | Treatment Time Side | 1 | 83 | 0.61 | 0.436 |
| Peak power (W) | Treatment | 1 | 83 | 0.24 | 0.627 |
| Peak power (W) | Time | 1 | 83 | 0.33 | 0.567 |
| Peak power (W) | Side | 1 | 83 | 84.81 | <0.001 |
| Peak power (W) | Period | 1 | 83 | 43.62 | <0.001 |
| Peak power (W) | Sequence | 1 | 11 | 1.79 | 0.208 |
| Peak power (W) | Treatment Time | 1 | 83 | 0.18 | 0.670 |
| Peak power (W) | Treatment Side | 1 | 83 | 2.11 | 0.150 |
| Peak power (W) | Time Side | 1 | 83 | 0.01 | 0.908 |
| Peak power (W) | Treatment Time Side | 1 | 83 | 0.09 | 0.766 |
| Time to peak torque (ms) | Treatment | 1 | 83 | 0.29 | 0.591 |
| Time to peak torque (ms) | Time | 1 | 83 | 0.13 | 0.721 |
| Time to peak torque (ms) | Side | 1 | 83 | 9.09 | 0.003 |
| Time to peak torque (ms) | Period | 1 | 83 | 2.33 | 0.131 |
| Time to peak torque (ms) | Sequence | 1 | 11 | 0.11 | 0.750 |
| Time to peak torque (ms) | Treatment Time | 1 | 83 | 0.21 | 0.648 |
| Time to peak torque (ms) | Treatment Side | 1 | 83 | 0.51 | 0.475 |
| Time to peak torque (ms) | Time Side | 1 | 83 | 4.72 | 0.033 |
| Time to peak torque (ms) | Treatment Time Side | 1 | 83 | 0.22 | 0.641 |
| Thigh pressure pain threshold (mmHg) | Treatment | 1 | 59 | 0.40 | 0.531 |
| Thigh pressure pain threshold (mmHg) | Time | 2 | 59 | 19.99 | <0.001 |
| Thigh pressure pain threshold (mmHg) | Period | 1 | 59 | 2.27 | 0.137 |
| Thigh pressure pain threshold (mmHg) | Sequence | 1 | 11 | 0.85 | 0.376 |
| Thigh pressure pain threshold (mmHg) | Treatment Time | 2 | 59 | 1.05 | 0.358 |
| Calf pressure pain threshold (mmHg) | Treatment | 1 | 59 | 0.13 | 0.725 |
| Calf pressure pain threshold (mmHg) | Time | 2 | 59 | 1.03 | 0.363 |
| Calf pressure pain threshold (mmHg) | Period | 1 | 59 | 1.02 | 0.317 |
| Calf pressure pain threshold (mmHg) | Sequence | 1 | 11 | 0.00 | 0.956 |
| Calf pressure pain threshold (mmHg) | Treatment Time | 2 | 59 | 0.94 | 0.396 |
| Thigh circumference (cm) | Treatment | 1 | 59 | 2.73 | 0.104 |
| Thigh circumference (cm) | Time | 2 | 59 | 2.17 | 0.123 |
| Thigh circumference (cm) | Period | 1 | 59 | 0.14 | 0.707 |
| Thigh circumference (cm) | Sequence | 1 | 11 | 0.10 | 0.760 |
| Thigh circumference (cm) | Treatment Time | 2 | 59 | 8.03 | <0.001 |
| Visual analogue scale score (mm) | Treatment | 1 | 83 | 3.73 | 0.057 |
| Visual analogue scale score (mm) | Time | 3 | 83 | 0.76 | 0.521 |
| Visual analogue scale score (mm) | Period | 1 | 83 | 2.24 | 0.139 |
| Visual analogue scale score (mm) | Sequence | 1 | 11 | 0.19 | 0.675 |
| Visual analogue scale score (mm) | Treatment Time | 3 | 83 | 0.51 | 0.678 |

Note. Participant was modelled as a random intercept. Fixed effects were treatment, time, period (stage), and sequence; side and its interactions were additionally included for bilateral knee isokinetic outcomes. Approx. p values are shown for descriptive reference only.
