# Supplementary material for: Selective recovery effects of short-term beetroot juice supplementation after exercise-induced muscle damage in trained female volleyball players
Source: J Int Soc Sports Nutr. 2026 Oct 20;23(1):2751785. doi: 10.1080/15502783.2026.2751785 (PMC13686210; doi:10.1080/15502783.2026.2751785)
Supplement: Supplementary file 2 [file RSSN_A_2751785_SM8395.docx]

**Supplementary_Table_S2_planned_posthoc_contrasts**

**Supplementary Table S2. Planned post hoc contrasts.**

| **Outcome** | **Side** | **Contrast** | **Estimate** | **95% CI** | **Adjusted P** | **Cohen's d** | **Magnitude** |
| --- | --- | --- | --- | --- | --- | --- | --- |
| CMJ height (cm) |  | 72 h post-EIMD vs baseline within BRJ | -0.89 | [-2.33, 0.54] | 0.254 | -0.60 | moderate |
| CMJ height (cm) |  | 72 h post-EIMD vs baseline within PLA | -2.49 | [-3.93, -1.05] | <0.001 | -1.67 | large |
| CMJ height (cm) |  | BRJ vs PLA at 72 h post-EIMD | 1.51 | [0.05, 2.97] | 0.033 | 1.01 | large |
| CMJ height (cm) |  | BRJ vs PLA at Baseline | -0.09 | [-1.55, 1.37] | 0.882 | -0.06 | trivial |
| Volleyball-specific agility test time (s) |  | 72 h post-EIMD vs baseline within BRJ | 0.17 | [-0.11, 0.45] | 0.420 | 0.58 | moderate |
| Volleyball-specific agility test time (s) |  | 72 h post-EIMD vs baseline within PLA | 0.31 | [0.03, 0.59] | 0.029 | 1.05 | large |
| Volleyball-specific agility test time (s) |  | BRJ vs PLA at 72 h post-EIMD | -0.04 | [-0.33, 0.24] | 0.834 | -0.15 | trivial |
| Volleyball-specific agility test time (s) |  | BRJ vs PLA at Baseline | 0.09 | [-0.19, 0.38] | 0.834 | 0.32 | small |
| Peak torque (Nm) | Left | 72 h post-EIMD vs baseline within BRJ (Left) | 1.84 | [-2.74, 6.41] | 1.000 | 0.43 | small |
| Peak torque (Nm) | Left | 72 h post-EIMD vs baseline within PLA (Left) | 0.48 | [-4.09, 5.06] | 1.000 | 0.11 | trivial |
| Peak torque (Nm) | Left | BRJ vs PLA at 72 h post-EIMD (Left) | -1.03 | [-5.64, 3.57] | 1.000 | -0.24 | small |
| Peak torque (Nm) | Left | BRJ vs PLA at Baseline (Left) | -2.39 | [-6.99, 2.22] | 1.000 | -0.55 | moderate |
| Peak torque (Nm) | Right | 72 h post-EIMD vs baseline within BRJ (Right) | 0.84 | [-3.73, 5.42] | 1.000 | 0.20 | trivial |
| Peak torque (Nm) | Right | 72 h post-EIMD vs baseline within PLA (Right) | 1.33 | [-3.25, 5.90] | 1.000 | 0.31 | small |
| Peak torque (Nm) | Right | BRJ vs PLA at 72 h post-EIMD (Right) | -2.10 | [-6.71, 2.50] | 1.000 | -0.49 | small |
| Peak torque (Nm) | Right | BRJ vs PLA at Baseline (Right) | -1.62 | [-6.23, 2.98] | 1.000 | -0.38 | small |
| Mean power (W) | Left | 72 h post-EIMD vs baseline within BRJ (Left) | -1.36 | [-7.00, 4.28] | 1.000 | -0.26 | small |
| Mean power (W) | Left | 72 h post-EIMD vs baseline within PLA (Left) | -0.46 | [-6.10, 5.19] | 1.000 | -0.09 | trivial |
| Mean power (W) | Left | BRJ vs PLA at 72 h post-EIMD (Left) | -2.62 | [-8.30, 3.06] | 1.000 | -0.49 | small |
| Mean power (W) | Left | BRJ vs PLA at Baseline (Left) | -1.71 | [-7.40, 3.97] | 1.000 | -0.32 | small |
| Mean power (W) | Right | 72 h post-EIMD vs baseline within BRJ (Right) | -2.02 | [-7.67, 3.62] | 1.000 | -0.38 | small |
| Mean power (W) | Right | 72 h post-EIMD vs baseline within PLA (Right) | 2.15 | [-3.49, 7.80] | 1.000 | 0.40 | small |
| Mean power (W) | Right | BRJ vs PLA at 72 h post-EIMD (Right) | -3.14 | [-8.82, 2.55] | 1.000 | -0.59 | moderate |
| Mean power (W) | Right | BRJ vs PLA at Baseline (Right) | 1.04 | [-4.64, 6.72] | 1.000 | 0.20 | trivial |
| Peak power (W) | Left | 72 h post-EIMD vs baseline within BRJ (Left) | -2.88 | [-21.85, 16.10] | 1.000 | -0.16 | trivial |
| Peak power (W) | Left | 72 h post-EIMD vs baseline within PLA (Left) | -1.97 | [-20.95, 17.00] | 1.000 | -0.11 | trivial |
| Peak power (W) | Left | BRJ vs PLA at 72 h post-EIMD (Left) | 0.86 | [-18.25, 19.97] | 1.000 | 0.05 | trivial |
| Peak power (W) | Left | BRJ vs PLA at Baseline (Left) | 1.77 | [-17.34, 20.87] | 1.000 | 0.10 | trivial |
| Peak power (W) | Right | 72 h post-EIMD vs baseline within BRJ (Right) | -4.16 | [-23.14, 14.81] | 1.000 | -0.23 | small |
| Peak power (W) | Right | 72 h post-EIMD vs baseline within PLA (Right) | 0.94 | [-18.04, 19.91] | 1.000 | 0.05 | trivial |
| Peak power (W) | Right | BRJ vs PLA at 72 h post-EIMD (Right) | -11.43 | [-30.54, 7.68] | 0.847 | -0.64 | moderate |
| Peak power (W) | Right | BRJ vs PLA at Baseline (Right) | -6.33 | [-25.44, 12.77] | 1.000 | -0.35 | small |
| Time to peak torque (ms) | Left | 72 h post-EIMD vs baseline within BRJ (Left) | -5.32 | [-16.71, 6.07] | 1.000 | -0.49 | small |
| Time to peak torque (ms) | Left | 72 h post-EIMD vs baseline within PLA (Left) | -5.37 | [-16.75, 6.02] | 1.000 | -0.50 | small |
| Time to peak torque (ms) | Left | BRJ vs PLA at 72 h post-EIMD (Left) | -3.40 | [-14.86, 8.07] | 1.000 | -0.32 | small |
| Time to peak torque (ms) | Left | BRJ vs PLA at Baseline (Left) | -3.44 | [-14.91, 8.03] | 1.000 | -0.32 | small |
| Time to peak torque (ms) | Right | 72 h post-EIMD vs baseline within BRJ (Right) | 1.88 | [-9.51, 13.26] | 1.000 | 0.17 | trivial |
| Time to peak torque (ms) | Right | 72 h post-EIMD vs baseline within PLA (Right) | 5.78 | [-5.60, 17.17] | 1.000 | 0.54 | moderate |
| Time to peak torque (ms) | Right | BRJ vs PLA at 72 h post-EIMD (Right) | -2.34 | [-13.81, 9.12] | 1.000 | -0.22 | small |
| Time to peak torque (ms) | Right | BRJ vs PLA at Baseline (Right) | 1.56 | [-9.90, 13.03] | 1.000 | 0.15 | trivial |
| Thigh pressure pain threshold (mmHg) |  | BRJ vs PLA at 24 h | 4.75 | [-3.32, 12.83] | 0.480 | 0.56 | moderate |
| Thigh pressure pain threshold (mmHg) |  | BRJ vs PLA at 48 h | 2.88 | [-5.20, 10.95] | 0.790 | 0.34 | small |
| Thigh pressure pain threshold (mmHg) |  | BRJ vs PLA at 72 h | -1.89 | [-9.97, 6.18] | 0.790 | -0.22 | small |
| Calf pressure pain threshold (mmHg) |  | BRJ vs PLA at 24 h | -9.73 | [-39.96, 20.50] | 1.000 | -0.30 | small |
| Calf pressure pain threshold (mmHg) |  | BRJ vs PLA at 48 h | 10.50 | [-19.73, 40.73] | 1.000 | 0.33 | small |
| Calf pressure pain threshold (mmHg) |  | BRJ vs PLA at 72 h | 12.12 | [-18.11, 42.34] | 1.000 | 0.38 | small |
| Thigh circumference (cm) |  | BRJ vs PLA at 24 h | -33.54 | [-69.28, 2.21] | 0.050 | -0.89 | large |
| Thigh circumference (cm) |  | BRJ vs PLA at 48 h | 26.16 | [-9.59, 61.90] | 0.081 | 0.69 | moderate |
| Thigh circumference (cm) |  | BRJ vs PLA at 72 h | 47.54 | [11.80, 83.28] | 0.004 | 1.26 | large |
| Thigh circumference (cm) |  | BRJ vs PLA at Baseline | 0.16 | [-0.47, 0.79] | 1.000 | 0.26 | small |
| Visual analogue scale score (mm) |  | BRJ vs PLA at 24 h | 0.18 | [-0.45, 0.81] | 1.000 | 0.28 | small |
| Visual analogue scale score (mm) |  | BRJ vs PLA at 48 h | 0.01 | [-0.62, 0.64] | 1.000 | 0.01 | trivial |
| Visual analogue scale score (mm) |  | BRJ vs PLA at 72 h | 0.44 | [-0.19, 1.07] | 0.326 | 0.69 | moderate |

Note. Holm-adjusted planned contrasts from the mixed-model framework. Immediate outcomes include between-condition comparisons at baseline and 72 h post-EIMD and within-condition baseline-versus-72 h contrasts. Recovery outcomes include between-condition comparisons at 24, 48, and 72 h post-EIMD. Standardized effect sizes are reported as model-based Cohen's d.
