# Supplementary material for: Selective recovery effects of short-term beetroot juice supplementation after exercise-induced muscle damage in trained female volleyball players
Source: J Int Soc Sports Nutr. 2026 Oct 20;23(1):2751785. doi: 10.1080/15502783.2026.2751785 (PMC13686210; doi:10.1080/15502783.2026.2751785)
Supplement: Supplementary file 3 [file RSSN_A_2751785_SM8424.docx]

**Supplementary_Table_S3_phase_specific_VAS_ThighPPT**

**Supplementary Table S3. Phase-specific descriptive statistics for visual analogue scale (VAS) and thigh pressure pain threshold.**

| **Outcome** | **Time** | **Phase 1 PLA (9)** | **Phase 1 BRJ (8)** | **Phase 2 PLA (5)** | **Phase 2 BRJ (8)** |
| --- | --- | --- | --- | --- | --- |
| Visual analogue scale score (mm) | 24 h | 37.05 ± 8.59 | 48.56 ± 6.72 | 38.96 ± 14.49 | 37.65 ± 6.06 |
| Visual analogue scale score (mm) | 48 h | 35.73 ± 10.15 | 35.60 ± 9.00 | 29.04 ± 11.39 | 35.17 ± 7.03 |
| Visual analogue scale score (mm) | 72 h | 31.10 ± 11.07 | 17.12 ± 5.53 | 18.56 ± 8.82 | 27.80 ± 3.92 |
| Thigh pressure pain threshold (mmHg) | 24 h | 181.62 ± 42.71 | 144.60 ± 40.36 | 135.60 ± 30.90 | 157.38 ± 23.79 |
| Thigh pressure pain threshold (mmHg) | 48 h | 154.25 ± 34.93 | 162.80 ± 31.89 | 136.00 ± 40.17 | 151.75 ± 23.96 |
| Thigh pressure pain threshold (mmHg) | 72 h | 143.88 ± 33.51 | 183.20 ± 40.61 | 183.60 ± 31.22 | 161.00 ± 33.79 |

Note. These descriptive statistics were re-extracted directly from the original SAV files for VAS and thigh pressure pain threshold. Values are mean ± SD. Phase-specific results are provided to complement the pooled mixed-model summaries in Table 4.
