# Supplementary material for: Selective recovery effects of short-term beetroot juice supplementation after exercise-induced muscle damage in trained female volleyball players
Source: J Int Soc Sports Nutr. 2026 Oct 20;23(1):2751785. doi: 10.1080/15502783.2026.2751785 (PMC13686210; doi:10.1080/15502783.2026.2751785)
Supplement: Supplementary file 4 [file RSSN_A_2751785_SM8438.docx]

**Supplementary_Table_S4_within_condition_change_effect_sizes**

**Supplementary Table S4. Within-condition change contrasts and effect sizes.**

| **Outcome** | **Treatment** | **Side** | **Contrast** | **Estimate** | **95% CI** | **Adjusted P** | **Cohen's d** | **Magnitude** |
| --- | --- | --- | --- | --- | --- | --- | --- | --- |
| Countermovement jump (CMJ) height (cm) | BRJ |  | Baseline vs 72 h post-EIMD | -2.49 | [-3.80, -1.18] | <0.001 | -1.67 | large |
| Countermovement jump (CMJ) height (cm) | PLA |  | Baseline vs 72 h post-EIMD | -0.89 | [-2.21, 0.42] | 0.127 | -0.60 | moderate |
| Volleyball-specific agility test time (s) | BRJ |  | Baseline vs 72 h post-EIMD | 0.31 | [0.05, 0.56] | 0.014 | 1.05 | large |
| Volleyball-specific agility test time (s) | PLA |  | Baseline vs 72 h post-EIMD | 0.17 | [-0.09, 0.43] | 0.140 | 0.58 | moderate |
| Peak torque (Nm) | BRJ | Left | Baseline vs 72 h post-EIMD | 0.48 | [-3.73, 4.70] | 1.000 | 0.11 | trivial |
| Peak torque (Nm) | BRJ | Right | Baseline vs 72 h post-EIMD | 1.33 | [-2.89, 5.54] | 1.000 | 0.31 | small |
| Peak torque (Nm) | PLA | Left | Baseline vs 72 h post-EIMD | 1.84 | [-2.38, 6.05] | 1.000 | 0.43 | small |
| Peak torque (Nm) | PLA | Right | Baseline vs 72 h post-EIMD | 0.84 | [-3.37, 5.06] | 1.000 | 0.20 | trivial |
| Mean power (W) | BRJ | Left | Baseline vs 72 h post-EIMD | -0.46 | [-5.66, 4.75] | 1.000 | -0.09 | trivial |
| Mean power (W) | BRJ | Right | Baseline vs 72 h post-EIMD | 2.15 | [-3.05, 7.36] | 1.000 | 0.40 | small |
| Mean power (W) | PLA | Left | Baseline vs 72 h post-EIMD | -1.36 | [-6.57, 3.84] | 1.000 | -0.26 | small |
| Mean power (W) | PLA | Right | Baseline vs 72 h post-EIMD | -2.02 | [-7.23, 3.18] | 1.000 | -0.38 | small |
| Peak power (W) | BRJ | Left | Baseline vs 72 h post-EIMD | -1.97 | [-19.46, 15.52] | 1.000 | -0.11 | trivial |
| Peak power (W) | BRJ | Right | Baseline vs 72 h post-EIMD | 0.94 | [-16.55, 18.43] | 1.000 | 0.05 | trivial |
| Peak power (W) | PLA | Left | Baseline vs 72 h post-EIMD | -2.88 | [-20.37, 14.61] | 1.000 | -0.16 | trivial |
| Peak power (W) | PLA | Right | Baseline vs 72 h post-EIMD | -4.16 | [-21.65, 13.33] | 1.000 | -0.23 | small |
| Time to peak torque (ms) | BRJ | Left | Baseline vs 72 h post-EIMD | -5.37 | [-15.88, 5.15] | 0.683 | -0.50 | small |
| Time to peak torque (ms) | BRJ | Right | Baseline vs 72 h post-EIMD | 5.78 | [-4.73, 16.30] | 0.683 | 0.54 | moderate |
| Time to peak torque (ms) | PLA | Left | Baseline vs 72 h post-EIMD | -5.32 | [-15.84, 5.20] | 0.683 | -0.49 | small |
| Time to peak torque (ms) | PLA | Right | Baseline vs 72 h post-EIMD | 1.88 | [-8.64, 12.39] | 0.683 | 0.17 | trivial |
| Thigh pressure pain threshold (mmHg) | BRJ |  | 24 h vs 48 h | -4.63 | [-13.34, 4.08] | 0.167 | -0.54 | moderate |
| Thigh pressure pain threshold (mmHg) | BRJ |  | 24 h vs 72 h | -11.51 | [-20.22, -2.80] | 0.003 | -1.35 | large |
| Thigh pressure pain threshold (mmHg) | BRJ |  | 48 h vs 72 h | -6.88 | [-15.59, 1.83] | 0.120 | -0.80 | large |
| Thigh pressure pain threshold (mmHg) | PLA |  | 24 h vs 48 h | -6.51 | [-15.22, 2.20] | 0.120 | -0.76 | moderate |
| Thigh pressure pain threshold (mmHg) | PLA |  | 24 h vs 72 h | -18.15 | [-26.86, -9.44] | <0.001 | -2.12 | large |
| Thigh pressure pain threshold (mmHg) | PLA |  | 48 h vs 72 h | -11.65 | [-20.36, -2.94] | 0.003 | -1.36 | large |
| Calf pressure pain threshold (mmHg) | BRJ |  | 24 h vs 48 h | -16.69 | [-49.30, 15.91] | 1.000 | -0.52 | moderate |
| Calf pressure pain threshold (mmHg) | BRJ |  | 24 h vs 72 h | -4.77 | [-37.37, 27.84] | 1.000 | -0.15 | trivial |
| Calf pressure pain threshold (mmHg) | BRJ |  | 48 h vs 72 h | 11.92 | [-20.68, 44.53] | 1.000 | 0.37 | small |
| Calf pressure pain threshold (mmHg) | PLA |  | 24 h vs 48 h | 3.54 | [-29.07, 36.14] | 1.000 | 0.11 | trivial |
| Calf pressure pain threshold (mmHg) | PLA |  | 24 h vs 72 h | 17.08 | [-15.53, 49.68] | 1.000 | 0.53 | moderate |
| Calf pressure pain threshold (mmHg) | PLA |  | 48 h vs 72 h | 13.54 | [-19.07, 46.14] | 1.000 | 0.42 | small |
| Thigh circumference (cm) | BRJ |  | 24 h vs 48 h | -10.38 | [-48.94, 28.17] | 1.000 | -0.27 | small |
| Thigh circumference (cm) | BRJ |  | 24 h vs 72 h | -22.23 | [-60.79, 16.33] | 0.536 | -0.59 | moderate |
| Thigh circumference (cm) | BRJ |  | 48 h vs 72 h | -11.85 | [-50.40, 26.71] | 1.000 | -0.31 | small |
| Thigh circumference (cm) | PLA |  | 24 h vs 48 h | 49.31 | [10.75, 87.86] | 0.004 | 1.30 | large |
| Thigh circumference (cm) | PLA |  | 24 h vs 72 h | 58.85 | [20.29, 97.40] | <0.001 | 1.56 | large |
| Thigh circumference (cm) | PLA |  | 48 h vs 72 h | 9.54 | [-29.02, 48.10] | 1.000 | 0.25 | small |
| Visual analogue scale score (mm) | BRJ |  | 24 h vs 48 h | 0.17 | [-0.48, 0.82] | 1.000 | 0.26 | small |
| Visual analogue scale score (mm) | BRJ |  | 24 h vs 72 h | 0.25 | [-0.40, 0.91] | 1.000 | 0.40 | small |
| Visual analogue scale score (mm) | BRJ |  | 48 h vs 72 h | 0.08 | [-0.57, 0.74] | 1.000 | 0.13 | trivial |
| Visual analogue scale score (mm) | PLA |  | 24 h vs 48 h | 0.18 | [-0.47, 0.84] | 1.000 | 0.29 | small |
| Visual analogue scale score (mm) | PLA |  | 24 h vs 72 h | 0.10 | [-0.55, 0.75] | 1.000 | 0.16 | trivial |
| Visual analogue scale score (mm) | PLA |  | 48 h vs 72 h | -0.08 | [-0.74, 0.57] | 1.000 | -0.13 | trivial |

Note. Within-condition change contrasts were derived from the same mixed-model framework. For recovery outcomes, a = 24 h vs 48 h, b = 24 h vs 72 h, and c = 48 h vs 72 h. For baseline/post outcomes, d = baseline vs 72 h post-EIMD. Standardized effect sizes are reported as model-based Cohen's d.
