# Supplementary material for: Selective recovery effects of short-term beetroot juice supplementation after exercise-induced muscle damage in trained female volleyball players
Source: J Int Soc Sports Nutr. 2026 Oct 20;23(1):2751785. doi: 10.1080/15502783.2026.2751785 (PMC13686210; doi:10.1080/15502783.2026.2751785)
Supplement: Supplementary file 5 [file RSSN_A_2751785_SM9699.docx]

**Supplementary Table S5. Sensitivity analyses for key functional and recovery outcomes in the unbalanced crossover dataset.**

| **Outcome** | **Primary contrast** | **Period P** | **Sequence P** | **Completers-only** | **Phase 1-only** | **Interpretation** |
| --- | --- | --- | --- | --- | --- | --- |
| Countermovement jump (CMJ) height (cm) | 1.51 [0.05, 2.97];  P = 0.033 | 0.012 | 0.741 | 1.51 [0.05, 2.97];  P = 0.033 | -0.02 [-4.71, 4.68];  P = 0.994 | Supported by the current completers-only crossover model; less precise in the smaller Phase 1-only subset. |
| Volleyball-specific agility test time (s) | -0.04 [-0.33, 0.24];  P = 0.834 | 0.819 | 0.865 | -0.04 [-0.33, 0.24];  P = 0.834 | -0.03 [-1.04, 0.99];  P = 0.957 | No clear treatment signal across the current primary and sensitivity analyses. |
| Visual analogue scale soreness (mm) | -1.89 [-9.97, 6.18];  P = 0.790 | 0.137 | 0.376 | -1.89 [-9.97, 6.18];  P = 0.790 | -13.98 [-24.13, -3.83]; P = 0.007 | No clear treatment signal in the primary completers-only model; the Phase 1-only estimate suggests phase sensitivity. |
| Thigh pressure pain threshold (mmHg) | 12.12 [-18.11, 42.34]; P = 1.000 | 0.317 | 0.956 | 12.12 [-18.11, 42.34];  P = 1.000 | 39.33 [-2.55, 81.20]; P = 0.066 | No clear treatment signal across the current primary and sensitivity analyses. |
| Calf pressure pain threshold (mmHg) | 47.54 [11.80, 83.28]; P = 0.004 | 0.707 | 0.760 | 47.54 [11.80, 83.28];  P = 0.004 | 53.25 [6.16, 100.34]; P = 0.027 | Supported by the current completers-only crossover model and the Phase 1-only sensitivity analysis. |
| Thigh circumference (cm) | 0.01 [-0.62, 0.64];  P = 1.000 | 0.139 | 0.675 | 0.01 [-0.62, 0.64];  P = 1.000 | -0.62 [-3.77, 2.53];  P = 0.700 | No clear treatment signal across the current primary and sensitivity analyses. |

**Note.** Primary contrast indicates BRJ vs PLA at 72 h post-EIMD for all outcomes. Period and Sequence P values were taken from the primary linear mixed model. Completers-only analyses were restricted to athletes who completed both crossover periods. Phase 1-only analyses were restricted to the first crossover period to reduce concern about attrition in Period 2 and potential crossover-related bias. P values in this table are unadjusted sensitivity estimates and are presented to assess robustness of the primary conclusions rather than to replace the prespecified primary analyses. Positive estimates favor BRJ for CMJ and pressure pain threshold outcomes, whereas negative estimates favor BRJ for agility time, soreness, and thigh circumference.
